# Clinical application of next generation sequencing-based haplotype linkage analysis in the preimplantation genetic testing for germline mosaicisms

**DOI:** 10.1186/s13023-023-02736-z

**Published:** 2023-06-03

**Authors:** Dongjia Chen, Yan Xu, Yu Fu, Yali Wang, Yuliang Liu, Chenhui Ding, Bing Cai, Jiafu Pan, Jing Wang, Rong Li, Jing Guo, Han Zhang, Yanhong Zeng, Xiaoting Shen, Canquan Zhou

**Affiliations:** 1 The First Affiliated Hospital, Sun Yat-Sen University, Guangzhou, 510080 China; 2 Guangdong Provincial Key Laboratory of Reproductive Medicine, Guangzhou, 510080 China; 3 The First Affiliated Hospital of Hainan Medical University, Hainan Medical University, Haikou, 570102 China; 4 Reproductive Medicine Center, The First Affiliated Hospital, Sun Yat-sen University, Guangzhou, 510080 China

**Keywords:** Germline mosaicism, Next generation sequencing, Single nucleotide polymorphism, Preimplantation genetic testing, Haplotype linkage analysis

## Abstract

**Background:**

Preimplantation genetic testing (PGT) for monogenic disorders (PGT-M) for germline mosaicism was previously highly dependent on polymerase chain reaction (PCR)-based directed mutation detection combined with linkage analysis of short tandem repeats (STRs). However, the number of STRs is usually limited. In addition, designing suitable probes and optimizing the reaction conditions for multiplex PCR are time-consuming and laborious. Here, we evaluated the effectiveness of next generation sequencing (NGS)-based haplotype linkage analysis in PGT of germline mosaicism.

**Methods:**

PGT-M with NGS-based haplotype linkage analysis was performed for two families with maternal germline mosaicism for an X-linked Duchenne muscular dystrophy (DMD) mutation (del exon 45–50) or an autosomal TSC1 mutation (c.2074C > T). Trophectoderm biopsy and multiple displacement amplification (MDA) were performed for a total of nine blastocysts. NGS and Sanger sequencing were performed in genomic DNA of family members and embryonic MDA products to detect DMD deletion and TSC1 mutation, respectively. Single nucleotide polymorphism (SNP) sites closely linked to pathogenic mutations were detected with NGS and served in haplotype linkage analysis. NGS-based aneuploidy screening was performed for all embryos to reduce the risk of pregnancy loss.

**Results:**

All nine blastocytes showed conclusive PGT results. Each family underwent one or two frozen-thawed embryo transfer cycles to obtain a clinical pregnancy, and the prenatal diagnosis showed that the fetus was genotypically normal and euploid for both families.

**Conclusions:**

NGS-SNP could effectively realize PGT for germline mosaicism. Compared with PCR-based methods, the NGS-SNP method with increased polymorphic informative markers can achieve a greater diagnostic accuracy. Further studies are warranted to verify the effectiveness of NGS-based PGT of germline mosaicism cases in the absence of surviving offsprings.

**Supplementary Information:**

The online version contains supplementary material available at 10.1186/s13023-023-02736-z.

## Introduction

Mosaicism refers to the presence of two or more cell lines with different genomic information in an individual, resulting from mutations during early embryonic development. Gonosomal mosaicism (mosaicism present in both somatic and gonadal tissues) is caused by mutations before primordial germ cell (PGC) differentiation [1]. After PGC differentiation, mutations can only lead to somatic or gonadal mosaicism. Germline mosaicism is the presence of both normal and mutated gametes as a result of gonosomal and gonadal mosaicism. Whole genome sequencing revealed that 3.8% of the mutations were mosaic in the parental germline [2]. Patients with germline mosaicism are often phenotypically normal, but are at a great risk of repeatedly giving birth to affected children [3, 4]. The first affected child of a parent with germline mosaicism is often misdiagnosed as a case of a de novo mutation, as germline mosaicism is often not suspected until the birth of a second affected child. The recurrence risk depends on whether the mosaic mutation is present in the paternal or maternal germline and the proportion of germ cells carrying the mutation [5]. The birth of a second affected child often poses a serious psychological and economic burden for families with germline mosaicism, especially for those with severe and poorly treated genetic diseases [4].

Duchenne muscular dystrophy (DMD), a terminal X-linked recessive hereditary muscular disease that affects one in every 3,500 live birth males [6], is characterized by symmetrical progressive muscle degeneration and weakness. Most DMD patients completely lose the ability to walk by the age of 12 years and die of respiratory and circulatory failure by the age of 20 years [7, 8]. DMD is caused by a mutation to the DMD gene (Xp21), which encodes the dystrophin protein. The DMD gene is relatively huge, consisting of 2.3 Mbp and 79 exons, and is prone to a high frequency of mutations, as one-third of DMD patients are sporadic cases [9]. Tuberous sclerosis (TSC), also known as Bourneville disease, is an autosomal dominant neurocutaneous syndrome with an incidence of 6.8–8.24 cases per 100,000 births [10]. TSC is characterized by facial angiofibroma, seizures, and mental retardation, and to a lesser extent, multisystemic damage [11]. The pathogenic genes of TSC are TSC1 (9q34) and TSC2 (16p13), which encode the tumor-suppressor proteins hamartin and tuberin, respectively. Approximately two-thirds of TSC patients carry de novo mutations [12]. At present, there is no effective treatment for DMD or TSC.

Once a germline mosaicism is suspected, invasive prenatal diagnosis combined with termination of the affected pregnancy is usually performed to avoid the birth of affected offspring. As an early form of prenatal diagnosis, preimplantation genetic testing (PGT) for monogenic diseases (PGT-M) can identify embryos free of genetic mutations before pregnancy, thereby effectively avoiding the mental anguish and physical pain associated with pregnancy termination. To reduce the impact of allele drop-out (ADO), current PGT-M for germline mosaicisms mainly relies on preimplantation genetic haplotyping (PGH), which is identifying at-risk chromosomes by the detection of gene markers closely linked to pathogenic mutations and screening for unaffected embryos without at-risk chromosomes [13–15]. Polymerase chain reaction (PCR)-based directed mutation detection combined with linkage analysis of short tandem repeats (STRs) has been applied to PGT-M for germline mosaicisms of TSC2 mutation [13, 14]. To date, there have been no reports of PGT-M for germline mosaicisms of DMD or TSC1 mutations.

Since the emergence of next generation sequencing (NGS), PGT technology has continued to advance. With the aid of target capture chips combined with high throughput sequencing, NGS can be applied for quick sequencing of pathogenic mutations and flanking single nucleotide polymorphisms (SNPs), which has greatly accelerated technical innovations for PGH [16–18]. Since SNPs account the largest number of polymorphic sites in the human genome, the accuracy of NGS-SNP diagnosis is superior to PCR-STR [19]. NGS-SNP has been successfully used in PGT of multiple monogenic diseases and matching of human leukocyte antigens [16–18, 20, 21]. When performing PGT-M for sporadic cases without a family history, NGS combined with SNP analysis of gametes and embryos can be employed for haplotype construction [22, 23]. However, there have been no reports of NGS applied to PGT-M for germline mosaicisms. Here PGT was successfully applied for germline mosaicisms of DMD and TSC1 mutations using the NGS approach.

## Results

### Preliminary study

**Fig. 1:**
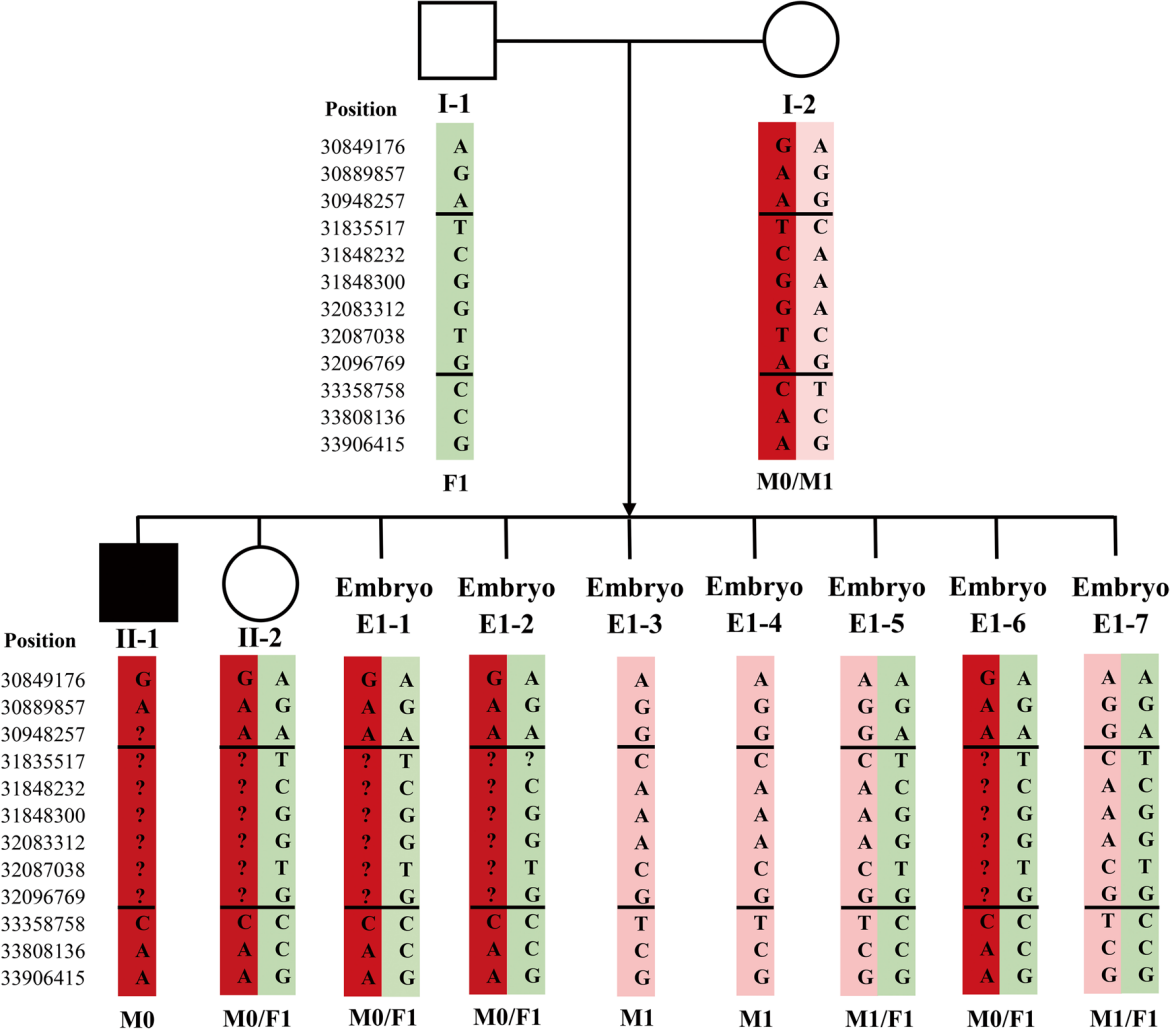
SNP-based haplotype linkage analysis of the *DMD* mutation (Family 1). The Figure displays only part of the SNP results. The double horizontal lines indicate the boundaries of the target *DMD* mutation. M0: maternal at-risk chromosome X. M1: maternal normal chromosome X. F1: paternal normal chromosome X

**Fig. 2:**
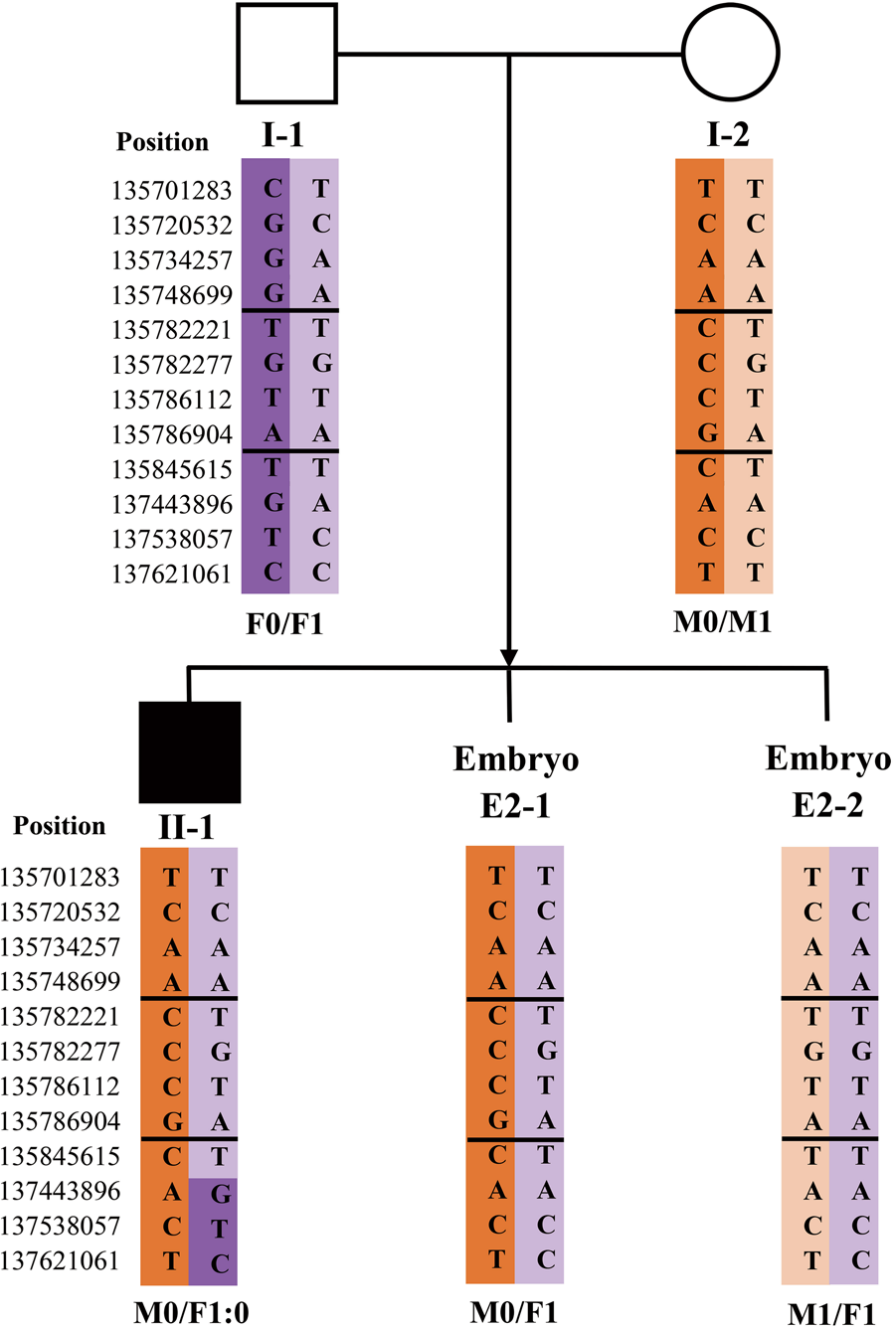
SNP-based haplotype linkage analysis of the *TSC1* mutation (Family 2). The Figure displays only part of the SNP results. The double horizontal lines indicate the boundaries of the target *TSC1* mutation. M0: maternal at-risk chromosome 9. M1: maternal normal chromosome 9. F0, F1: paternal normal chromosome 9

Informative SNPs were used to identify haplotypes of the two families. The number of informative SNPs is shown in Table 1. For Family 1, the mother with gonadal mosaicism, affected son, and carrier daughter shared the same haplotype through which the at-risk chromosome for the *DMD* mutation was confirmed (Fig. 1). For Family 2, the mother with gonosomal mosaicism shared the same haplotype with the affected son, by which the at-risk chromosome for the *TSC1* mutation was confirmed (Fig. 2).

**Table 1:** Number of informative/selected SNP**s**

Target region	Upstream		Within the target region	Downstream	
	Within 1 Mb	Outside of 1 Mb		Within 1 Mb	Outside of 1 Mb
DMD	16/23	–	(29–45)/104^a^	12/23	–
TSC1	37/50	3/10	–	35/50	10/10

^a^ A total of 104 single nucleotide polymorphisms (SNPs) within the *DMD* gene were selected. The number of informational SNPs in each embryo varied between 29 and 45

### Ovum pick-up cycle

Clinical ovum pick-up was performed for both families. For Family 1, nine oocytes were retrieved and normally fertilized. Of the nine oocytes, seven were cultured into blastocysts and successfully biopsied. For Family 2, a total of six oocytes were retrieved. Of the six oocytes, five were normally fertilized and developed into day 3 embryos. Only two blastocysts were formed and were selected for trophectoderm biopsy.

### PGT cycle

#### Family 1

For Family 1, the PGT results were conclusive for all seven obtained embryos (Table 2). Of the seven embryos, five were female and two were male (Table 3). Haplotype linkage analysis showed that three female embryos carried the at-risk chromosome (Fig. 1), the remaining two female and two male embryos did not carry the at-risk chromosome. NGS-based DMD mutation detection confirmed that none of the seven embryos was affected (Additional file 1: Table S1).

**Table 2:** Summary of the PGT results of Family 1

Sample	Embryo sexing	PGT-M (*DMD* del exon 45–50)		PGT-A	Transferable
		PGH	NGS		
Wife	Female	M0/M1	Normal	–	–
Husband	Male	F1	Normal	–	–
Son	Male	M0	Affected	–	–
Daughter	Female	M0/F1	Carrier	–	–
E1-1	Female	M0/F1	Carrier	Normal	Required genetic counselling before transfer
E1-2	Female	M0/F1	Carrier	Normal	
E1-3	Male	M1	Normal	+ mos(13)(q34)(49%)	
E1-4	Male	M1	Normal	Normal	√
E1-5	Female	M1/F1	Normal	+ mos(2)(q37.3)(61%)	×
E1-6	Female	M0/F1	Carrier	Complex mosaicism in low percentage	×
E1-7	Female	M1/F1	Normal	+ mos(4)(p 16.2)(55%)	×

*PGH* Preimplantation genetic haplotyping. *PGT-M* Preimplantation genetic testing for monogenic diseases. *PGT-A* Preimplantation genetic testing for aneuploidy. M0: maternal at-risk chromosome X. M1: maternal normal chromosome X. F1: paternal normal chromosome X. Normal: noncarrier. √: transferable embryo. × : untransferable embryo

**Table 3:** Next generation sequencing-based embryo sexing of chromosome Y for Family 1

Name	Start	End	Depth						
			E1-1	E1-2	E1-3	E1-4	E1-5	E1-6	E1-7
Y-1	2,655,295	2,655,296	4	0	12,928	6200	1	3	7
Y-2	2,656,695	2,656,696	0	0	117	57	0	0	0
Y-3	6,635,044	6,635,045	0	0	296	160	0	0	0
Y-4	7,072,004	7,072,005	0	0	504	1440	0	1	0
Y-5	8,212,251	8,212,252	0	0	1	1	0	0	0
Y-6	14,829,485	14,829,486	1	1	1556	2010	1	1	1
Y-7	15,995,908	15,995,909	0	0	413	261	0	1	1
Y-8	17,920,297	17,920,298	0	0	611	585	0	0	0
Y-9	19,418,831	19,418,832	0	0	464	550	2	0	0
Y-10	23,282,952	23,282,953	4	1	2953	5044	1	0	1
Result			Female	Female	Male	Male	Female	Female	Female

Preimplantation genetic testing for aneuploidy (PGT-A) indicated that only three of the seven embryos were euploids. Of the remaining four embryos, two were mosaic embryos with high aneuploid percentage (≥ 50%), one was a mosaic embryo with low aneuploid percentage (< 50%), and one was identified with complex mosaic aneuploidy (i.e., mosaic aneuploidy involves multiple chromosomes).

Therefore, only one unaffected euploid embryo was obtained for Family 1 (E1-4), which could be transferred in a subsequent frozen-thawed embryo transfer (FET) cycle. E1-1 and E1-2 were euploid carrier embryos, whereas E1-3 was an unaffected embryo with a low percentage of mosaic aneuploidy (Table 2). So, the transfer E1-1, E1-2, and E1-3 was not recommended, and genetic counseling was suggested prior to transplantation. E1-5, E1-6, and E1-7 were unaffected or carrier embryos, but none were deemed transferable because of a high percentage of mosaic or complex mosaic aneuploidy (Table 2).

Family 1 underwent first FET cycle with E1-4 in August 2020, which did not result in pregnancy. After sufficient genetic counseling, they underwent second FET cycle with E1-3 in December 2020 and achieved clinical pregnancy. An amniocentesis test was conducted and showed a normal fetal genotype with no chromosomal abnormalities. Finally, Family 1 gave birth to a healthy liveborn baby in September 2021.

#### Family 2

**Fig. 3:**
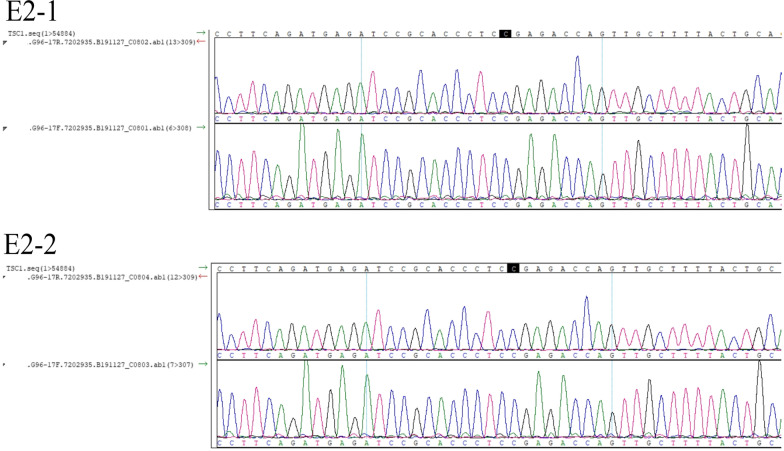
Sanger sequencing showed that neither embryo of Family 2 carried the *TSC1* mutation

For Family 2, the PGT results of two embryos were conclusive (Table 4). Haplotype linkage analysis indicated that one of two (E2-1) carried the at-risk chromosome (Fig. 2), while Sanger sequencing showed that both embryos carried the normal TSC1 gene (Fig. 3). PGT-A showed that both embryos were euploid.

**Table 4:** Summary of the PGT results of Family 2

Sample	PGT-M (*TSC1* c.2074C > T)		PGT-A	Transferable
	PGH	Sanger sequencing		
Wife	M0/M1	–	–	–
Husband	F0/F1	–	–	–
Son	M0/F1:0	–	–	–
E2-1	M0/F1	Normal	Euploid	Requiring required genetic counselling before transfer
E2-2	M1/F1	Normal	Euploid	√

*PGH* Preimplantation genetic haplotyping. *PGT-A* Preimplantation genetic testing for aneuploidy. M0: maternal at-risk chromosome 9. M1: maternal normal chromosome 9. F0, F1: paternal normal chromosome 9. Normal: noncarrier. √: transferable embryo

Finally, Family 2 obtained a euploid embryo (E2-2) free of the TSC1 mutation, which was deemed transferable. E2-1 carried the at-risk chromosome, but no TSC1 mutation was detected by Sanger sequencing. Thus, transfer of E2-1 was not recommended and genetic counseling was suggested prior to transfer of this embryo.

Finally, Family 2 underwent E2-2 transfer into the uterus in August 2020, which resulted in clinical pregnancy. Moreover, they underwent an amniocentesis test and found that the fetus exhibited a normal genotype with no chromosomal abnormalities. Finally, Family 2 gave birth to a healthy liveborn baby in July 2021.

## Discussion

Patients with germline mosaicisms have a more torturous pregnancy history than those without. Many patients do not understand the severity of germline mosaicisms and still insist on obtaining normal offspring through natural pregnancy, which leads to repeated induced labors, as experienced by Family 2 in this study. By eliminating affected embryos before the establishment of pregnancy, PGT-M greatly reduces the time to achieve healthy offspring and, thus, is a better choice for patients with germline mosaicism.

The accuracy of diagnosis can be affected by ADO if PGT-M is only based on direct mutation detection. Especially for dominant genetic diseases, this approach may result in affected embryos being mistakenly transferred. Therefore, PGT-M for germline mosaicism is largely dependent on PGH, which can improve the accuracy of diagnosis close to 100% [24]. Previous PGT-M for germline mosaicism was highly dependent on direct mutation detection and indirect STR-based haplotype linkage analysis (3–12 STRs were detected) through PCR methods, which achieved satisfactory PGT results [13–15]. However, as the number of STRs is usually limited, the impact of ADO cannot be completely eliminated. Also, the design of suitable probes and optimization of the reaction conditions for multiplex PCR are time-consuming and laborious. As compared with PCR, the high throughput, high coverage, and high sensitivity features are great advantages to NGS for the identification of mosaicism [25]. The results of the present study suggest that NGS-based PGH can effectively realize PGT for the detection of germline mosaicism. In addition, more than 100 SNPs were selected for each family, which can compensate the shortcomings of STR-based linkage analysis.

In this study, as we found an X-linked recessive disease in Family 1 and a genomic DNA (gDNA) mosaicism in the wife of Family 2, we inferred that the at-risk chromosomes in both families were of maternal origin. In addition, both families had surviving offsprings, through which the at-risk haplotypes were clearly identified. However, for certain gonadal mosaicism cases associated with autosomal genetic diseases, when a limited number of offspring is insufficient to determine the origin of at-risk chromosomes, it is likely to lead to misdiagnosis, as reported by Patel et al. [15] and Viart et al. [26]. In this case, the possibility of germline mosaicism with a somatic component should first be excluded by making full use of somatic samples, such as blood, buccal epithelial cells, saliva, fingernails, etc. [15, 25]. When the origin of the at-risk chromosome cannot be determined by analysis of somatic cells, the mystery can be solved by detecting sperms or polar bodies [26], which is especially suitable for patients with no offspring. Our group and others have previously reported that for PGT-M without a family history, haplotype analysis can still be realized by NGS-SNP using gametes [23, 27, 28]. Therefore, it is reasonable that gamete-based haplotype analysis via NGS-SNP can also be applied for cases of gonadal mosaicism when it is difficult to determine the origin of at-risk chromosomes.

During PGT-M for germline mosaicism, the results of indirect haplotype linkage analysis may occasionally conflict with those of direct mutation detection, such as E2-1 of Family 2 in the present study. Explaining this conflict to the family is very important during genetic counseling. A possible reason for such conflicting results is the presence of both affected and unaffected cell lines in the gonads of the parents. Therefore, this embryo might result from a normal gamete that carried an at-risk chromosome but no pathogenic mutation. A second possible reason is a false negative result caused by ADO during whole genome amplification (WGA) [29]. Although thorough analyses of adequate coverage NGS can show minor alleles, the interference of ADO on the diagnosis cannot be completely excluded. Therefore, it is not recommended to transfer embryos with at-risk chromosomes, even if there are no apparent pathogenic mutations. This approach, however, may lead to the waste of potentially unaffected embryos. If the patient insists on the transfer of such embryos, it is necessary to fully inform the patient of the risks and the necessity of prenatal diagnosis during pregnancy.

Notably, our study is not the first to report the use of NGS-based PGT-M in germline Mosaicisms, as in May 2020, Hu et al. reported the application of NGS-PGT-M in four families with maternal mosaicism which resulted in four healthy babies [30]. However, our study deserves attention for the following reasons: First of all, our study and Hu et al.’s study were carried out independently in different reproductive centers at the same time, but both studies obtained the same result, that the transmission of mosaic variants could be effectively prevented by NGS-PGT-M [30]. Secondly, the number of affected children/fetuses of the families included in Hu et al. 's study ranged from 0 to 2 [30]. However, we reported a more severe case: a total of four children/fetuses with the DMD mosaic mutation were conceived in Family 2. These patients did not seek PGT-M treatment in time, possibly because they were not aware of the effectiveness of NGS-PGT-M for germline mosaicism. Therefore, our study may help popularize the feasibility of NGS-PGT-M for germline mosaicism and help more families with germline mosaicism obtain healthy offspring. Thirdly, we are the first to observe that during NGS-PGT-M for germline mosaicism, the results of indirect haplotype linkage analysis might occasionally conflict with those of direct mutation detection. Undoubtedly, germline mosaicism will increase the complexity of PGT. Therefore, the clinician should carefully interpret the results by identifying the origin of at-risk chromosomes and provide the patients with more detailed explanations and genetic counseling.

## Conclusions

NGS-SNP could effectively realize PGT for germline mosaicism. Compared with PCR-based methods, the NGS-SNP method with increased polymorphic informative markers can achieve a greater diagnostic accuracy. Further studies are warranted to verify the effectiveness of NGS-based PGT of germline mosaicism cases in the absence of surviving offsprings.

## Materials and methods

### Patients

The study protocol was approved by the Research Ethics Committee of the First Affiliated Hospital of Sun Yat-sen University (Guangzhou, China) and conducted in accordance with the tenets of the Declaration of Helsinki. In addition, written informed consent was obtained from all patients prior to study inclusion.

#### Family 1

A DMD male was born to 22-year-old parents in 2010. Genetic testing of the infant revealed a deletion to the exon 45–50 region of the DMD gene. In 2011, a healthy female was born. Subsequent genetic testing showed that the second infant was a carrier of a DMD mutation. In 2015 and 2016, the wife underwent two rounds of induced labor due to a prenatal diagnosis of fetal DMD. Mutation detection of peripheral blood samples collected from both parents and all four grandparents showed that all had normal DMD genotypes and euploid karyotypes. Due to suspected maternal gonadal mosaicism, the couple requested PGT at our center.

#### Family 2

**Fig. 4:**
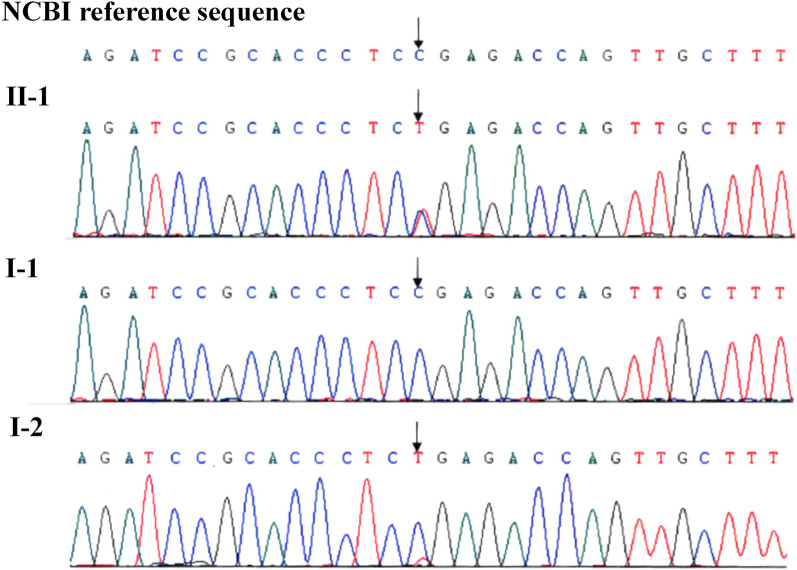
Sanger sequencing revealed that the wife of Family 2 exhibited mosaicism for the TSC1 mutation. II-1, the affected boy. I-1, the husband. I-2, the wife

In 2016, a male infant with TSC was born to a 32-year-old husband and a 30-year-old wife. Genetic testing of the infant revealed a heterozygous mutation to exon 17 of the TSC1 gene (c.2074C > T, p.Arg692*). Sanger sequencing revealed that the husband of Family 2 lacked the TSC1 mutation, while the wife exhibited mosaicism for the TSC1 mutation, with a 10–15% mosaic ratio. The mosaic ratio was calculated from Sanger sequencing peaks (Fig. 4). The karyotypes of the couple were normal. The wife was diagnosed with gonosomal mosaicism and referred to our center for PGT.

### Embryo preparation and trophectoderm biopsy

Female patients underwent a standard long-term pituitary down-regulation protocol for controlled ovulation. After intramuscular injection of 10,000 U of human chorionic gonadotropin, oocytes were retrieved under the guidance of B-ultrasound 36 h later and fertilized by intracytoplasmic sperm injection. The resulting embryos were cultured to the blastocyst stage. Blastocysts with average embryo quality were selected for laser-assisted trophectoderm biopsy, as described in our previous report [21]. After biopsy, the blastocysts were timely vitrified with the use of a Cryotop® Vitrification kit (Kitazato Biopharma Co., Ltd., Fuji, Japan).

### DNA sample preparation

The trophectoderm samples were subjected to MDA using the REPLI-g Single Cell Kit (Qiagen, Hilden, Germany). The QIAamp DNA Blood Maxi Kit (Qiagen) was used to extract gDNA from the peripheral blood of all parents and their infants.

### Mutation detection and NGS-based haplotype linkage analysis

For Family 1, 37 primers were designed in the exon 45–50 region of the DMD gene and the affected samples could be identified when the NGS sequencing depth of these corresponding amplicons was 0 (Additional file 1: Table S1). Moreover, 10 specific sites on the Y chromosome were selected for embryo sexing (Table 3). For Family 2, the mutation of the *TSC1* gene (NM 000368.4 chr9: 135,766,735–135,820,094) was detected using Sanger sequencing with a BigDye Terminator Cycle Sequencing Kit version 3.1 (Applied Biosystems Inc., Foster City, CA) in an ABI 3130 Genetic Analyzer (Applied Biosystems Inc.).

For NGS-based haplotype construction, 104 SNPs within the *DMD* gene (NM 004006.2 chrX: 31,137,345–33,357,726) and 46 SNPs within a 1-Mbp flanking region were selected for Family 1, and 120 SNPs flanking the *TSC1* gene were selected for Family 2. The Ion AmpliSeq™ designer tool was used to design all primers in this study. After DNA purification, a cDNA library was constructed and enriched. The MDA products underwent NGS using the MiSeqDx instrument (Illumina). In a preliminary study, the gDNA of all parents and their infants were tested for the above-mentioned mutation sites and selected SNPs with the same procedures to identify at-risk chromosomes.

For all embryonic MDA samples, after library construction (VeriSeq PGS-MiSeq kit, Illumina), NGS-based aneuploidy screening was performed using the MiSeq Reagent Kit v3-PGS (Illumina) on a MiSeq instrument (Illumina).

The data obtained in this study were analyzed by Peking Jabrehoo Med Tech Co., Ltd. (Beijing, China).

### Frozen-thawed embryo transfer cycle and prenatal diagnosis

In each FET cycle, a transferable blastocyst was selected under adequate genetic counseling and subsequently transferred into the uterus of the female patient under the guidance of transvaginal ultrasound. The blood human chorionic gonadotropin level was detected 14 days after embryo transfer to confirm the biochemical pregnancy. At 5–6 weeks of gestation, clinical pregnancy was confirmed when the gestational sac and fetal heartbeat could be detected using ultrasound. At 18–20 weeks of gestation, amniotic fluid was obtained by amniocentesis for prenatal diagnosis to determine the fetal genotype and screen for chromosome aneuploidy.

## Supplementary Information

**Additional file 1:** Results of the next generation sequencing-based DMD mutation detection for Family 1.

## Data Availability

The data of this study are either included in the article (or in its Additional file 1: Results of the next generation sequencing-based DMD mutation detection for Family 1) or available from the corresponding author on reasonable request. The data are not publicly available due to privacy or ethical restrictions.

## Abbreviations

PGC: Primordial germ cell
DMD: Duchenne muscular dystrophy
TSC: Tuberous sclerosis
PGT: Preimplantation genetic testing
PGT-M: Preimplantation genetic testing for monogenic diseases
ADO: Allele drop-out
PGH: Preimplantation genetic haplotyping
PCR: Polymerase chain reaction
STR: Short tandem repeats
NGS: Next generation sequencing
SNP: Single nucleotide polymorphism
MDA: Multiple displacement amplification
gDNA: Genomic DNA
FET: Frozen-thawed embryo transfer
WGA: Whole genome amplification
