# Supplementary material for: Clinical application of next generation sequencing-based haplotype linkage analysis in the preimplantation genetic testing for germline mosaicisms
Source: Orphanet J Rare Dis. 2023 Jun 3;18:137. doi: 10.1186/s13023-023-02736-z (PMC10239584; doi:10.1186/s13023-023-02736-z)
Supplement: Supplementary file 1 — Additional file 1: Results of the next generation sequencing-based DMD mutation detection for Family 1. [file 13023_2023_2736_MOESM1_ESM.docx]

**Supplementary Table 1. Results of the next generation sequencing-based *DMD* mutation detection for Family 1.**

| **No.** | **Chromosome** | **Start** | **End** | **I-1** | **I-2** | **II-1** | **II-2** | **E1-1** | **E1-2** | **E1-3** | **E1-4** | **E1-5** | **E1-6** | **E1-7** |
| --- | --- | --- | --- | --- | --- | --- | --- | --- | --- | --- | --- | --- | --- | --- |
| 1 | chrX | 31835469 | 31835616 | 166 | 123 | 0 | 95 | 39 | 31 | 121 | 37 | 65 | 42 | 54 |
| 2 | chrX | 31838030 | 31838252 | 1603 | 1038 | 0 | 1092 | 264 | 158 | 562 | 225 | 355 | 197 | 331 |
| 3 | chrX | 31843680 | 31843897 | 189 | 160 | 0 | 94 | 56 | 47 | 144 | 73 | 76 | 55 | 84 |
| 4 | chrX | 31848189 | 31848410 | 820 | 543 | 0 | 563 | 1487 | 959 | 2599 | 1755 | 1903 | 1204 | 1672 |
| 5 | chrX | 31854775 | 31854999 | 401 | 300 | 0 | 208 | 584 | 497 | 872 | 878 | 825 | 419 | 775 |
| 6 | chrX | 31863147 | 31863371 | 752 | 501 | 0 | 369 | 170 | 130 | 325 | 373 | 227 | 141 | 241 |
| 7 | chrX | 31867550 | 31867769 | 667 | 581 | 0 | 437 | 407 | 381 | 585 | 714 | 537 | 330 | 636 |
| 8 | chrX | 31873271 | 31873466 | 1066 | 754 | 0 | 580 | 404 | 412 | 680 | 844 | 506 | 455 | 619 |
| 9 | chrX | 31879652 | 31879877 | 1 | 0 | 0 | 0 | 1 | 1 | 5 | 10 | 3 | 2 | 2 |
| 10 | chrX | 31887675 | 31887880 | 1939 | 1250 | 0 | 1274 | 566 | 517 | 1230 | 1701 | 1051 | 631 | 1097 |
| 11 | chrX | 31893130 | 31893346 | 208 | 137 | 0 | 113 | 32 | 46 | 93 | 141 | 112 | 55 | 93 |
| 12 | chrX | 31893335 | 31893511 | 226 | 176 | 0 | 192 | 64 | 45 | 179 | 233 | 128 | 76 | 152 |
| 13 | chrX | 31896501 | 31896724 | 310 | 277 | 0 | 188 | 137 | 181 | 547 | 595 | 465 | 187 | 353 |
| 14 | chrX | 31901282 | 31901504 | 9589 | 6439 | 2 | 5523 | 1672 | 2122 | 7247 | 6223 | 5414 | 1971 | 4759 |
| 15 | chrX | 31905410 | 31905619 | 521 | 455 | 0 | 293 | 426 | 585 | 2111 | 1899 | 1677 | 639 | 1548 |
| 16 | chrX | 31920934 | 31921132 | 107 | 93 | 0 | 79 | 20 | 9 | 36 | 47 | 35 | 10 | 16 |
| 17 | chrX | 31927062 | 31927278 | 220 | 224 | 0 | 119 | 81 | 110 | 301 | 291 | 202 | 59 | 236 |
| 18 | chrX | 31947688 | 31947889 | 199 | 169 | 0 | 140 | 79 | 171 | 464 | 594 | 300 | 73 | 481 |
| 19 | chrX | 31948511 | 31948732 | 1077 | 837 | 0 | 607 | 467 | 905 | 2557 | 2519 | 1392 | 307 | 2162 |
| 20 | chrX | 31950037 | 31950228 | 358 | 311 | 0 | 198 | 110 | 210 | 552 | 665 | 329 | 89 | 541 |
| 21 | chrX | 31950217 | 31950388 | 406 | 362 | 0 | 234 | 173 | 368 | 1096 | 1231 | 614 | 167 | 894 |
| 22 | chrX | 31952723 | 31952925 | 1319 | 729 | 1 | 795 | 602 | 1088 | 2963 | 3268 | 1916 | 510 | 2678 |
| 23 | chrX | 31967424 | 31967618 | 77 | 72 | 0 | 64 | 6 | 24 | 37 | 17 | 36 | 14 | 31 |
| 24 | chrX | 31971057 | 31971282 | 2718 | 1907 | 0 | 1406 | 1165 | 2419 | 3598 | 2773 | 3428 | 1353 | 3128 |
| 25 | chrX | 31983053 | 31983270 | 188 | 195 | 0 | 95 | 71 | 109 | 180 | 115 | 173 | 61 | 182 |
| 26 | chrX | 31986341 | 31986530 | 224 | 250 | 0 | 173 | 145 | 208 | 295 | 197 | 363 | 117 | 428 |
| 27 | chrX | 31986519 | 31986636 | 651 | 501 | 0 | 307 | 208 | 289 | 470 | 359 | 482 | 128 | 414 |
| 28 | chrX | 31993574 | 31993788 | 171 | 114 | 0 | 118 | 101 | 116 | 234 | 155 | 241 | 77 | 261 |
| 29 | chrX | 32014790 | 32014936 | 193 | 177 | 0 | 114 | 98 | 159 | 350 | 374 | 271 | 150 | 299 |
| 30 | chrX | 32020119 | 32020343 | 569 | 432 | 0 | 365 | 305 | 347 | 904 | 929 | 702 | 465 | 865 |
| 31 | chrX | 32077846 | 32078074 | 4112 | 3317 | 1 | 2048 | 3942 | 4629 | 8626 | 7222 | 9324 | 6447 | 9640 |
| 32 | chrX | 32083203 | 32083421 | 293 | 244 | 0 | 207 | 160 | 248 | 344 | 379 | 365 | 286 | 427 |
| 33 | chrX | 32086960 | 32087180 | 749 | 582 | 0 | 424 | 326 | 508 | 679 | 849 | 868 | 579 | 1024 |
| 34 | chrX | 32090064 | 32090290 | 572 | 418 | 0 | 415 | 353 | 532 | 861 | 918 | 894 | 416 | 1108 |
| 35 | chrX | 32096660 | 32096878 | 2966 | 2232 | 0 | 1396 | 810 | 1550 | 2129 | 2098 | 2292 | 859 | 2410 |
| 36 | chrX | 32112259 | 32112431 | 200 | 160 | 0 | 131 | 69 | 67 | 104 | 126 | 119 | 51 | 151 |
| 37 | chrX | 32125977 | 32126205 | 1393 | 1085 | 0 | 791 | 1356 | 1337 | 2170 | 1728 | 2169 | 463 | 2245 |

In the case of Family 1, 37 primers were designed for the exon 45–50 region of the *DMD* gene. The affected samples, such as II-1, could be identified when the next-generation sequencing (NGS) depth of these corresponding amplicons was 0. The NGS-based DMD mutation detection confirmed that none of the seven embryos was affected.

DMD: X-linked Duchenne muscular dystrophy
